# Risk assessment of deoxynivalenol in high-risk area of China by human biomonitoring using an improved high throughput UPLC-MS/MS method

**DOI:** 10.1038/s41598-018-22206-y

**Published:** 2018-03-01

**Authors:** Chunli Deng, Chenglong Li, Shuang Zhou, Xiaodan Wang, Haibin Xu, Dan Wang, Yun Yun Gong, Michael N. Routledge, Yunfeng Zhao, Yongning Wu

**Affiliations:** 1 China National Center for Food Safety Risk Assessment, Key laboratory of Food Safety Risk Assessment, Ministry of Health, Beijing, 100021 PR China; 2 School of Food Science and Nutrition, University of Leeds, Leeds, LS2 9JT UK; 3 School of Medicine, University of Leeds, Leeds, LS2 9JT UK

## Abstract

A risk assessment of deoxynivalenol (DON) was recently conducted for the residents in Henan province, China, where wheat as the staple food are highly consumed. A high-throughput sensitive UPLC-MS/MS method following 96-well μElution solid-phase extraction (SPE) were developed and validated for the determination of DON biomarkers in human urine. Isotope labelled internal standard, ^13^C-DON, was used for accurate quantification. Urinary samples collected from 151 healthy Chinese aged 2–78 years were processed with and without enzyme hydrolysis to determine total and free biomarkers, respectively. DON, and de-epoxy-deoxynivalenol (DOM-1) to a lesser extent, can be frequently detected in these samples both with and without enzyme hydrolysis. Free DOM-1 was detected at low level in human urine for the first time. Total DON was detected in all samples with a mean concentration at 47.6 ng mL^−1^. The mean and median probable daily intakes (PDI) for the whole participants, estimated to be 1.61 μg/kg bw and 1.10 μg/kg bw, both exceeded the PMTDI (1 μg/kg bw/day), indicating a potential risk for the residents in this area, especially for children and adolescents.

## Introduction

Deoxynivalenol (DON), belonging to the trichothecenes group produced by *Fusarium spp*. is one of the most prevalent mycotoxins^1^. Various crops are easily infested by these molds and thereby contaminated with DON, which is responsible for gastro-intestinal problems in humans as well as other potential adverse effects on human health, including immunosuppression and impairment of reproduction and development^2^. In response, the Joint FAO/WHO Expert Committee on Food Additives (JECFA) conducted a series of risk assessments and established a provisional maximum tolerable daily intake (PMTDI) of 1 μg/kg bw/day for the total amount of DON and its acetylated derivatives, 3-acetyl-deoxynivalenol (3ADON) and 15-acetyl-deoxynivalenol (15ADON)^3^. This led to many countries setting maximum permitted levels for DON^4^.

After ingestion, DON undergoes a rapid metabolization to DON-glucuronide conjugates, mainly DON-3-glucuronide (D-3-GlcA) and deoxynivalenol-15-glucuronide (D-15-GlcA)^5^. Additionally, a small proportion of DON can be detoxified to DOM-1 by gut microbiota, which can also conjugate with glucuronic acid and be excreted in the urine^6^.

Humans and animals are exposed to DON by ingestion of contaminated food^1^. Assessment of human exposure to mycotoxins is conventionally performed by analysis of food contamination levels and calculation of intake based on consumption data^7–9^. The heterogeneous distribution of mycotoxins in food may affect the accuracy of these results, whereas a biomarkers-based strategy can provide a less biased measure of mycotoxin intake.

A strong correlation has been demonstrated between the total DON (free DON together with DON-glucuronides) in urine and dietary DON intake in several studies^5,10,11^. DON biomarker analysis was initiated by Meky *et al*., via the measurement of total DON after enzymatic deconjugation^12^ and further improved by inclusion of isotope internal standard correction^13^. Subsequently, a number of approaches for the determination of DON biomarkers have been proposed for biological samples, involving gas chromatography-mass spectrometry (GC-MS)^14–17^, liquid chromatography-mass spectrometry (LC-MS)^13,18–20^, liquid chromatography-tandem mass spectrometry (LC-MS/MS)^6,21–24^, and other rapid screening methods such as Fourier-Transform Infrared Spectrometry^25^, fluorescence excitation-emission matrix^26^, and enzyme-linked immunosorbent assay (ELISA)^27^. Among them, LC-MS/MS provides excellent accuracy, sensitivity and selectivity, and has become a preferred technique. Most of these studies focused only on DON, DOM-1 and their glucuronides. DON acetylated derivatives (3-A-DON and 15-A-DON), also known as masked DON^28^, were commonly not included in the biological sample analysis, since these compounds could be digested to release DON *in vivo*. In these assays, solid-phase extraction (SPE) and immunoaffinity columns have been widely used for sample preparation, effectively reducing matrix interference and achieving a high increase in sensitivity. However, the labor-intensive and time-consuming steps of these conventional approaches present challenges to their further application in large-scale analysis.

To address such issues, we have developed a high-throughput UPLC-MS/MS method involving a 96-well μElution plate for the determination of total and free DON and DOM-1 in urine. This is the first application of μElution plate for DON biomarker analysis, which enables the simultaneous preparation of multiple samples without evaporation and reconstitution steps. Using this method, 151 urine samples collected from healthy volunteers in Henan province, China were evaluated for DON exposure.

## Results

### Method development and validation

**Figure 1:**
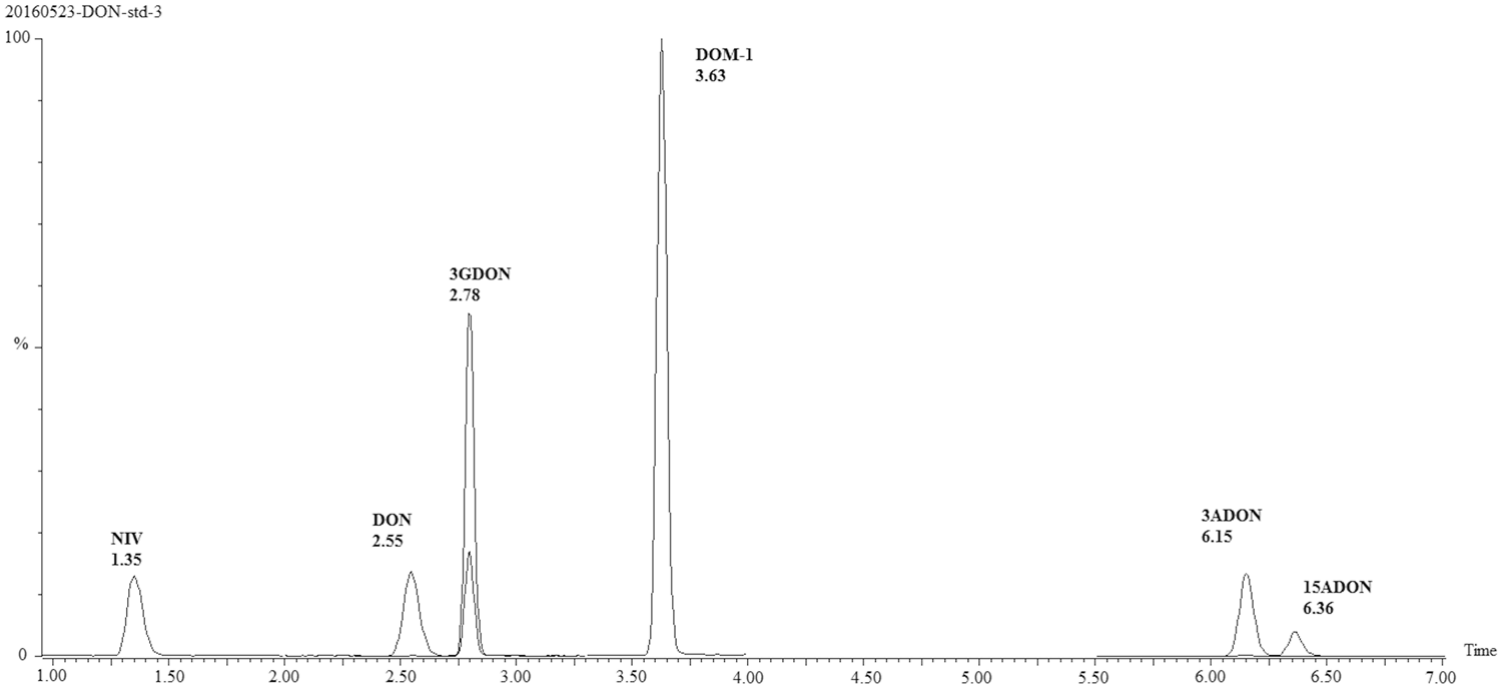
Extracted ion chromatograms based on MRM transitions for DON, DOM-1 and their major analogs (5 ng mL^−1^ of each compound).

MS/MS conditions were optimized manually by individual infusions of each analyte standard. The detailed parameters for each analyte were optimized as summarized in Table 1. Waters CORTECS C18 UPLC column (2.1 mm × 100 mm, 1.6 μm) with methanol and water as mobile phase under a gradient elution provided a complete separation of the analytes as well as their analogs for a single run, as displayed in Fig. 1. It is noteworthy that formic acid and ammonium formate used as mobile phase additives resulted in a strong suppression of ionization and thereby worse signal intensity of DON and DOM-1. A high-throughput sample preparation strategy implementing a 96-well Oasis® PRiME HLB μElution Plate was used for DON biomarker analysis. To improve recovery and selectivity, the detailed parameters associated with loading, washing and elution buffer as well as the enzymatic hydrolysis process were optimized (see Supplementary Information, Table S1 and Figure S1). To our best knowledge, this is the first report that enables high-throughput analysis for DON biomarkers, allowing 96 urine samples (one plate) to be processed within 2 h with good extraction recoveries (88.3~112%) and well-controlled matrix interference (67.4~83.2%).

**Table 1:** MRM transitions of the analytes.

Analyte	Parent ion (m/z)	Daughter ion (m/z)	Cone voltage (V)	Collision energy (eV)
DON	297.2	231.2	+36	+10
		249.2^a^	+36	+8
^13^C-DON	312.2	245.1	+20	+10
		263.2^a^	+20	+8
DOM-1	281.2	109.1	+26	+14
		233.2^a^	+26	+8

^a^Transition used for quantification.

Method validation was carried out as described in the Method section, following the recommendations of EMEA^29^ and US FDA^30^. The LOD and LOQ were 0.5 ng mL^−1^ and 1 ng mL^−1^, respectively for DON. For DOM-1, the LOD and LOQ were 0.1 ng mL^−1^ and 0.2 ng mL^−1^, respectively (Table 2). All analytes showed good linearity from their respective LOQ up to 100 ng mL^−1^, with correlation coefficients (R^2^) fell between 0.9920 and 0.9999. DON and DOM-1 displayed excellent method recoveries (R_M_) ranged from 80% to 112%. The inter-day and intra-day RSD were 3.8–12.5% and 3.2–13.3%. And no apparent carry-over was observed by injections of reagent blanks directly after high contaminated urine samples. A summary of validation parameters can be found in Table 2, all in accordance with the acceptance criteria.

**Table 2:** Sensitivity, accuracy and precision of the developed method.

Analyte	Spiked level (ng mL^−1^)	Measured (ng mL^−1^)	Method recovery (%)	RSD_r_		LOD (ng mL^−1^)	LOQ (ng mL^−1^)
				Intra-day (n = 6)	Inter-day (n = 18)		
DON	2	1.9	94	12.5	13.3	0.5	1
	10	11.2	112	3.8	3.2		
	50	20.8	104	7.1	8.4		
DOM-1	2	1.6	80	7.3	9.5	0.1	0.2
	10	9.1	91	6.4	4.5		
	50	18.2	91	4.4	6.6		

### Urinary DON biomarkers in the Chinese subjects

The high-throughput strategy was implemented to monitor the occurrence of DON biomarkers in urine samples collected from 151 healthy volunteers in Henan province, China. The demographic characteristics of the subjects are shown in Table 3.

**Table 3:** Characteristics of the subjects (mean ± SD (range)).

Variables	Children	Adolescents	Adults	elderly
Number of subjects	33	14	68	36
Male	2	2	34	18
Female	31	12	34	18
Age	6 ± 3 (2–12) years	15 ± 1 (13–17) years	40 ± 13 (21–64) years	72 ± 4 (66–81) years
Weight	25 ± 10 (12–50) kg	51 ± 8 (40–64) kg	70 ± 13 (45–95) kg	65 ± 11 (46–91) kg

**Figure 2:**
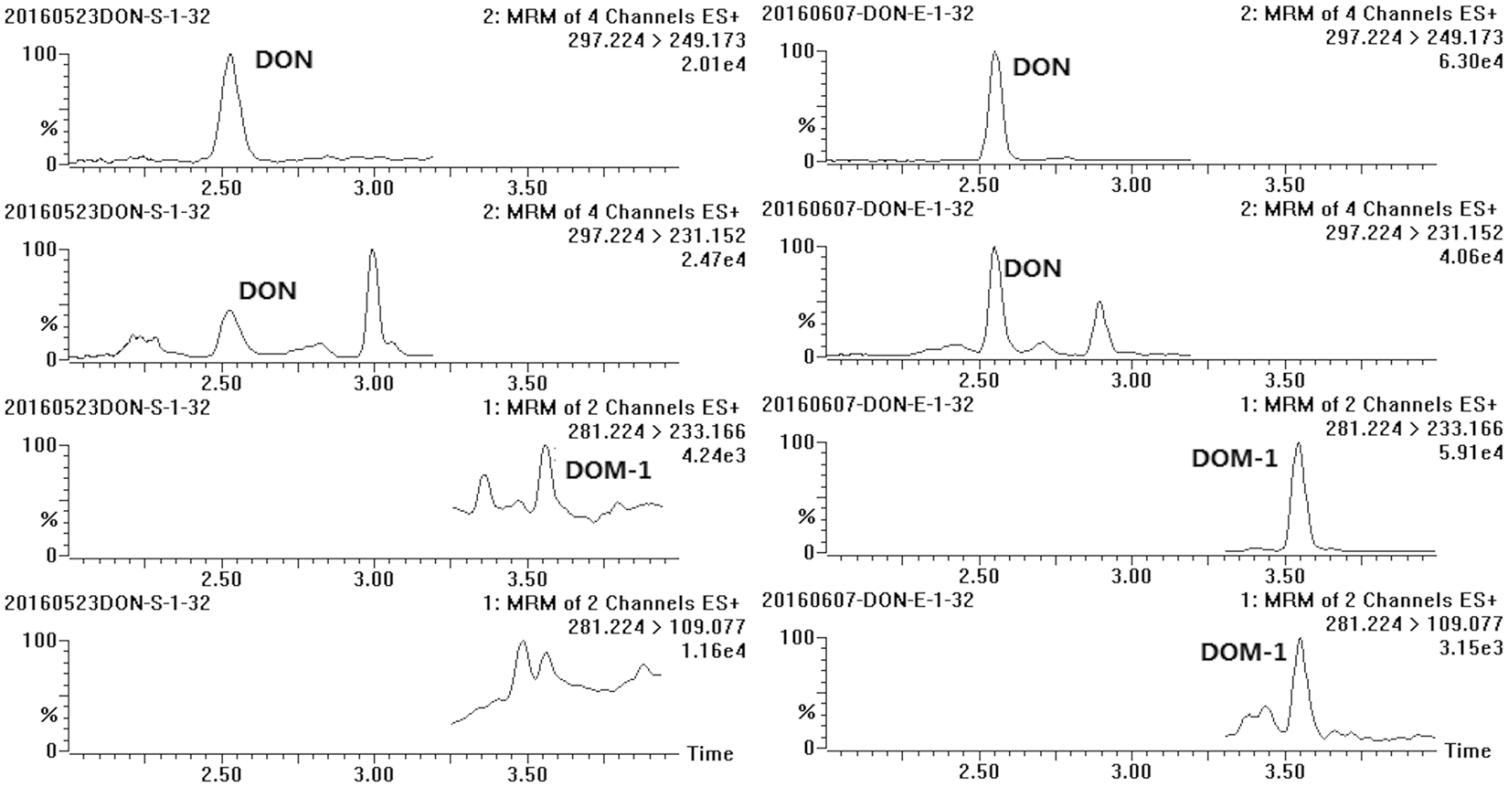
Chromatograms of a naturally contaminated urine sample before (left) and after (right) β-glucuronidase hydrolysis (free DON, 20.4 ng mL^−1^; free DOM-1, <LOQ; total DON, 116.2 ng mL^−1^; total DOM-1, 6.4 ng mL^−1^).

Chromatograms of a human urine sample naturally contaminated with DON and DOM-1 are shown in Fig. 2. In the absence of the β-glucuronidase digestion step, 92.7% (n = 140/151) of samples were positive for free DON (fDON) and 2.0% (n = 3/151) were positive for free DOM-1 (fDOM-1). The mean level (range) of fDON was 8.25 (<LOD-47.0) ng mL^−1^. Free DOM-1 was quantified in only one sample at a low level of 0.23 ng mL^−1^. After enzymatic hydrolysis, urinary total DON (tDON) was quantified in 100% (n = 151/151) of the samples, with the mean value (range) of 47.6 (1.36–247) ng mL^−1^; and a detection rate (30.5%, n = 46/151) of total DOM-1 (tDOM-1) was obtained, at a mean level (range) of 0.28 (<LOD-6.43) ng mL^−1^ (Table 4). As can be seen, glucuronide conjugates are the main metabolites for both DON and DOM-1. It should also be mentioned that, DON acetylated derivatives (3-A-DON and 15-A-DON), known as masked DON were also measured in our study. The key experimental parameters were presented in Supplementary Information (Tables S2 and S3). However, neither of them was detected in 151 urine samples (both with and without enzyme hydrolysis), which provide further demonstration for the rapid digestion of 3-A-DON and 15-A-DON *in vivo* after ingestion.

**Table 4:** Summary of free and total DON and it’s metabolites in 151 urine samples.

Compound	Positive n (%)	Mean (±SD) (ng mL^−1^)	Median (ng mL^−1^)	Range (ng mL^−1^)
fDON	140 (92.7)	8.25 (±8.74)	5.48	ND–47.0
tDON	151 (100)	47.6 (±49.2)	32.5	1.36–247
fDOM-1	3 (2.0)	0.052 (±0.015)	0.05	ND–0.23
tDOM-1	46 (30.5)	0.28 (±0.79)	0.05	ND–6.43

ND, level below LOD; positive samples refer to the levels higher than LOD.

When calculating the mean and median values, level below LOQ was set to half of the LOQ, and level below LOD was set to half of the LOD.

The urinary tDON (free DON + DON-glucuronides) concentrations taken as biomarker for exposure to DON were further analyzed by gender and 4 age groups (0–12, 13–18, 19–65, and >65). The mean level of tDON was slightly higher in female (52.8 ± 56.5 ng mL^−1^) than in male (38.8 ± 32.2 ng mL^−1^), but the difference did not reach statistical significance (P = 0.475). All the 4 age groups were positive for DON and DOM-1. The mean levels of tDON were highest in children (age ≤ 12, 63.2 ± 52.6 ng mL^−1^) and adolescents (age 13–18, 73.1 ± 61.0 ng mL^−1^), with no significant difference (P = 0.664) between them. Urinary tDON was about 1.5-fold lower in adults (age 19–65, 45.1 ± 44.5 ng mL^−1^) than in children and adolescents (P < 0.05). The elderly group (age > 65, 27.8 ± 42.2 ng mL^−1^) had the lowest tDON levels (P < 0.01), as presented in Table 5.

**Table 5:** Urinary tDON and tDOM-1 by gender and age groups.

	Compound	Positive	Mean (± SD)	Median	Range
		n (%)	ng mL^−1^	ng mL^−1^	ng mL^−1^
Gender	Male, n = 56				
	tDON	56 (100%)	38.8 (±32.2)	29.8	3.11–132
	fDON	49 (87.5%)	5.96 (±6.03)	4.25	ND–25.2
	tDOM-1	14 (25.0%)	0.37 (±1.08)	0.05	ND–6.43
	Female, n = 95				
	tDON	95 (100%)	52.8 (±56.5)	33.4	1.36–247
	fDON	91 (95.8%)	9.60 (±9.78)	6.47	ND–47.0
	tDOM-1	32 (33.7%)	0.22 (±0.54)	0.05	ND–3.49
Age	Age ≤ 12, n = 33				
	tDON	33 (100%)	63.2 (±52.6)	44.6	7.42–224
	fDON	32 (97.0%)	11.3 (±10.5)	7.79	ND–47.0
	tDOM-1	12 (36.4%)	0.16 (±0.25)	0.05	ND–1.05
	12 < Age ≤ 18, n = 14				
	tDON	14 (100%)	73.1 (±61.0)	52.4	11.4–240
	fDON	14 (100%)	13.0 (±10.8)	9.85	1.00–37.4
	tDOM-1	5 (35.7%)	0.11 (±0.10)	0.05	ND–0.35
	18 < Age ≤ 65, n = 68				
	tDON	68 (100%)	45.1 (±44.5)	29.8	3.31–213
	fDON	65 (95.6%)	8.05 (±7.38)	5.79	ND–31.1
	tDOM-1	21 (30.9%)	0.36 (±1.03)	0.05	ND–6.43
	Age > 65, n = 36				
	tDON	36 (100%)	27.8 (±42.2)	10.98	1.36–247
	fDON	29 (80.6%)	3.96 (±6.49)	2.48	ND–37.0
	tDOM-1	8 (22.2%)	0.40 (±0.74)	0.05	ND–2.91

ND, level below LOD; positive samples refer to the levels higher than LOD.

When calculating the mean and median values, level below LOQ was set to half of the LOQ, and level below LOD was set to half of the LOD.

## Discussion

### Urinary DON levels comparison

The present study was conducted for the residents in Henan province located in the central part of China, where wheat as the staple food, are consumed often at higher levels than in other provinces in China^31^. Accordingly, the average urinary concentration of tDON in the healthy subjects was much higher than those from Shanghai (n = 60, 97% positive, mean 4.8 ng mL^−1^)^20^ and Yunnan (n = 4, 100% positive, mean 12 ng mL^−1^) inhabitants^12^, and slightly higher than those of cancer patients in Henan province (100% positive, mean 37 ng mL^−1^) in 2003^12^. This reflected regional and temporal variability of DON exposure in the Chinese population.

Moreover, the tDON values in this study were also higher than those reported in Bangladesh (n = 54, 52% positive, mean 0.86 ng mL^−1^; n = 62, 27% positive, mean 0.17 ng mL^−1^)^21,22^, Cameroon (n = 220, 73% positive, mean 2.22 ng mL^−1^; n = 145, 43% positive, mean 5.93 ng mL^−1^)^32,33^, Egypt (n = 93, 68% positive, mean 1.11 ng/mg creatinine)^34^, Nigeria (n = 120, 5% positive, mean 3.9 ng mL^−1^)^35^, Tanzania (n = 166, 51% positive, mean 2.5 ng mL^−1^)^10^, South Africa (n = 53, 100% positive, mean 20.4 ng/mg creatinine)^36^, Austria (n = 27, 59% positive, mean 20.4 ng mL^−1^)^37^, France (n = 67, 99% positive, 0.5–28.8 ng mL^−1^)^38^, Germany (n = 50, 100% positive, mean 9.02 ng mL^−1^; n = 30, 100% positive, mean 7.15 ng mL^−1^; n = 101, 30% positive, mean 3.38 ng mL^−1^)^22,39,40^, Italy (n = 52, 96% positive, mean 11.89 ng mL^−1^; n = 10, 70% positive, mean 3.7 ng mL^−1^)^41,42^, Spain (n = 54, 69% positive, mean 23.3 ng/mg creatinine)^16^, Sweden (n = 29, 97% positive, mean 10.8 ng mL^−1^; n = 326, 90% positive, mean 2.9 ng mL^−1^; n = 252, 73% positive, mean 5.38 ng mL^−1^)^43–45^, U.K. (n = 15, 100% positive, mean 13.5 ng mL^−1^; n = 25, 100% positive, mean 10.8 ng mL^−1^; n = 300, 99% positive, mean 8.9 ng/mg creatinine; n = 35, 100% positive, mean 11.6 ng mL^−1^; n = 34, 68% positive, mean 2.4 ng mL^−1^; n = 85, 100% positive, mean 10.3 ng/mg creatinine)^6,13,46–49^ and Haiti (n = 142, 17% positive, mean 3.2 ng mL^−1^)^23^. On the other hand, the determined tDON levels in the Chinese participants was lower than those in Belgian volunteers (n = 32, 100% positive, mean 59.0 ng mL^−1^)^24^ and pregnant women (n = 40) from Croatia (97.5% positive, mean 111.8 ng/mL, range 4.8–1238 ng/mg)^50^.

### Urinary DOM-1 levels comparison

Urinary tDOM-1 after β-glucuronidase hydrolysis was reported in the low ng mL^−1^ range in several studies^6,16,22,24,34,39,45,49^, whereas it was not detected in other surveys^37,46^. In our study, tDOM-1 was detected in 30.5% of the samples, comparable with the prevalence of tDOM-1 in France (34%)^38^, Belgium (25%)^24^, Germany (40%; 50%)^22,39^ and UK (37% in 2012 and 40% in 2013)^6^, but apparently higher than in Sweden (8%)^45^, Spain (3.7%)^16^, Egypt (2%)^34^ and another study in UK (3%)^49^.

Considering the occurrence of tDOM-1 in several previous studies, fDOM-1 in urine can also be anticipated. In our study, fDOM-1 was first evidenced in human urine, which was detected in 2.0% (n = 3/151) of the samples. All the three samples were from male adults; their urinary tDON, tDOM-1 and fDOM-1 levels were 20.7 ng mL^−1^, 4.07 ng mL^−1^ and 0.22 ng mL^−1^, 42.0 ng mL^−1^, 6.43 ng mL^−1^and <LOQ, 32.2 ng/ml, 2.53 ng mL^−1^ and <LOQ, respectively. It is noteworthy that the three positive samples also shared high levels of tDOM-1 (ranked of 1^st^, 2^nd^ and 7^th^), whereas their tDON levels were not remarkably high (ranked 58^th^, 77^th^ and 100^th^). It is possible that these participants are more likely to detoxify DON to DOM-1 than others. This is the first demonstration of urinary free DOM-1 in humans.

### Correlation between different urinary biomarkers

**Figure 3:**
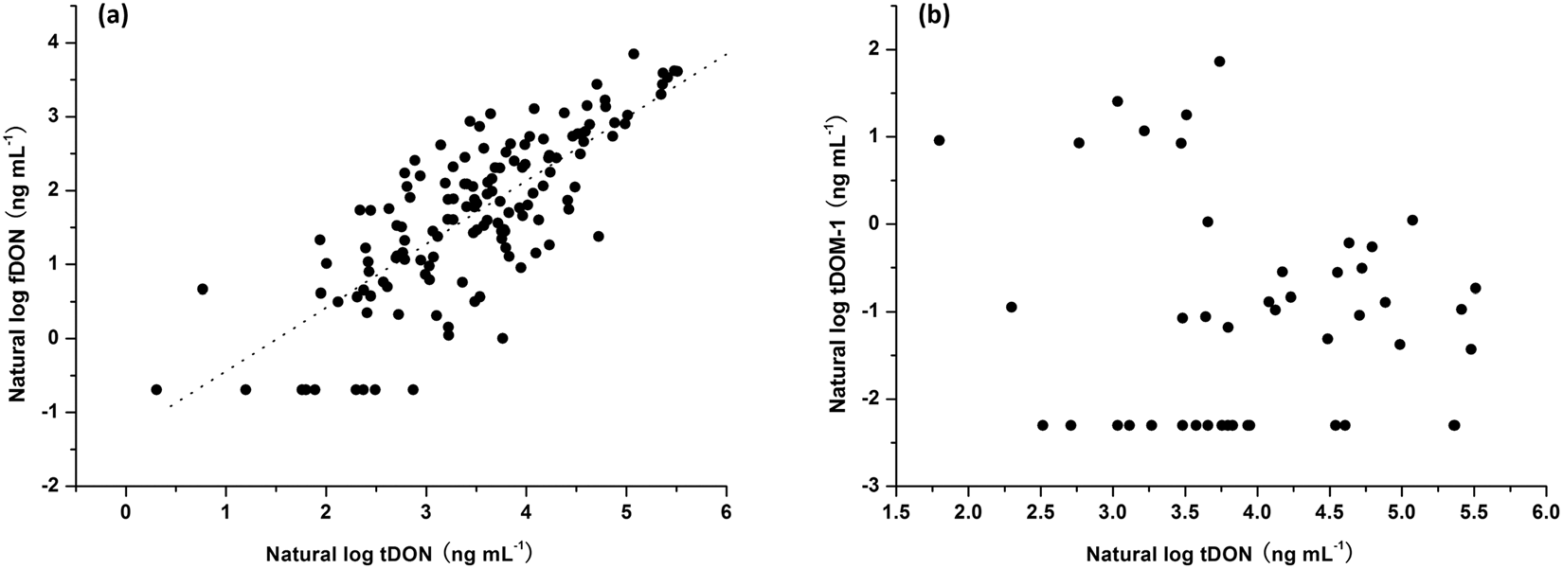
Scatterplot of urinary fDON (**a**) and urinary tDOM-1 (**b**) against urinary tDON, all in logarithmic scale. Levels below LOQ were set to half of the LOQ, and levels below LOD were set to half of the LOD.

Free DON levels were significantly correlated with the levels of tDON (Fig. 3a, r = 0.765, P < 0.001). On average 82.7% of the tDON was present as DON-glucuronides, in line with the recent findings in Austria (86%) directly quantifying DON, D-3-GlcA and D-15-GlcA^37^, and in UK (91.1%) comparing DON levels before and after β-glucuronidase hydrolysis^49^. Conjugation with glucuronic acid to DON-glucuronides appears to be a major route of DON detoxification and excretion. These results confirm that tDON is a more important urinary biomarker than fDON in reflecting the dietary DON exposure.

On the contrary, no significant correlation existed between tDOM-1 and tDON (Fig. 3b, r = −0.013, P = 0.932) for samples with detectable tDOM-1. However, the level of tDON (75.7 ± 65.6 ng mL^−1^, p < 0.001) was higher for those samples positive for tDOM-1 compared to the samples where tDOM-1 was not detected (35.3 ± 33.7 ng mL^−1^). In the 46 positive samples urinary tDOM-1 represented 3.17% (range 0.05–19.6%) of the amount of urinary tDON. Five samples among them possessed proportions of tDOM-1 (10.5–19.6% of tDON) higher than 10%.

### Estimated dietary DON intake

A probable daily intake (PDI) for DON could be estimated for the participants using equation (1), based on the urinary DON biomarker levels determined in this study and a urinary excretion rate published before^5^:

1 $${\rm{PDI}}=\frac{C\times V\times 100}{W\times E}\,$$

where *C* = total DON concentration (μg L^−1^), *V* = daily urine excretion (L), *W* = the individual body weight of each participant (kg), *E* = excretion rate (%).

A mean daily urine excretion was assumed to be 0.5 L for children and 1.5 L for adults^51,52^. An excretion rate of 68% (including 52% as DON-glucuronides and 16% as free DON)^5^ was used for the calculation. PDI calculated for DON ranged 0.038–9.62 μg/kg bw; and 79 of the 151 participants (52.3%) exceeded the PMTDI value set by JECFA (1 μg/kg bw/day)^3^. For the 4 age groups, children (2.09 ± 1.81 μg/kg bw) and adolescents (3.08 ± 2.44 μg/kg bw) have the highest PDI, with no significant difference (P = 0.126) between them. The PDI of DON was lower (P < 0.05) for adults (1.41 ± 1.47 μg/kg bw) than for children and adolescents. The elderly group (0.98 ± 1.41 μg/kg bw) had the lowest PDI (P < 0.01), as summarized in Table 6. Remarkably, the mean and median PDI for the entire cohort estimated to be 1.61 μg/kg bw and 1.10 μg/kg bw both exceeded the PMTDI, indicating a potential risk for the residents in Henan province, China. This could be partially attributed to the high consumption of cereals in this area.

**Table 6:** PDI of DON by 4 age groups.

Age	Mean (± SD)	Median	Range	Exceeding PMTDI
	μg/kg bw	μg/kg bw	μg/kg bw	n (%)
Age ≤ 12, n = 33	2.09 (±1.81)	1.47	0.23–7.57	23 (69.7%)
12 < Age ≤ 18, n = 14	3.08 (±2.44)	2.28	0.58–9.62	12 (85.7%)
18 < Age ≤ 65, n = 68	1.41 (±1.47)	0.99	0.12–9.26	34 (50%)
Age > 65, n = 36	0.98 (±1.41)	0.54	0.038–7.79	10 (27.8%)
Total, n = 151	1.61 (±1.73)	1.10	0.038–9.62	79 (52.3%)

When calculating the PDI, level below LOQ was set to half of the LOQ, and level below LOD was set to half of the LOD.

This situation was similar for Croatia (mean 111.8 ng/mL)^50^ and Belgain (mean 59.0 ng mL^−1^)^24^, mean daily intakes being 4.1 and 2.2 μg/kg bw/d respectively, exceeding the PMTDI. Especially in Croatia, nearly half of the subjects were estimated to exceed the PMTDI. On the contrary, most other studies around the world reported acceptable mean levels of tDON ranging from 0.2 to 20 ng/mL as mentioned above, corresponding to the daily intakes between 0.007 and 0.74 μg/kg bw/d, below the PMTDI value of 1 μg/kg bw/d.

## Methods

### Chemicals and materials

Standard solutions of DON (100 μg mL^−1^), DOM-1 (50 μg mL^−1^), and ^13^C_15_-DON (10 μg mL^−1^) were purchased from Biopure (Tulln, Austria). Beta-glucuronidase (Type IX from *E. coli*) was from Sigma-Aldrich (MO, USA). LC-MS grade water, acetonitrile, methanol, formic acid and ammonia acetate were supplied by Fisher Scientific (Leicestershire, United Kingdom). All other chemicals and reagents used were of analytical grade or better. The Oasis PRiME HLB 96-well μElution plate (3 mg/30 μm) was product of Waters (Milford, MA, USA). A mixed standard containing 10 μg mL^−1^ of each analyte was prepared in ACN/H_2_O (50/50, v/v) and stored at 4 °C. Working dilutions of mixed standards were freshly prepared for each run in methanol/H_2_O (20/80). The enzyme solution containing 2000 U mL^−1^ β-glucuronidase was prepared in phosphate buffer (0.075 mol L^−1^, pH 6.8) freshly on each day of use.

### Sample collection and storage

Urine samples of 151 healthy volunteers aged 2–78 years (56 males, 95 females) were collected in Henan province located in the middle of China. For each person, morning urine samples were collected on three consecutive days, immediately frozen stored at −70 °C. The urine from the three days were mixed at a 1:1:1 ratio to make one sample prior to DON biomarker analyses. The study was approved by the ethics committee of China National Center for Food Safety Risk Assessment, and all the methods were performed according to the approved guidelines and regulations. All the participants were completely informed of the purpose of this study, and the informed consents from the adult participants or parents on behalf of their children who participated the study were obtained.

### Preparation of calibration standards and quality control samples

Serial calibration standard solutions at levels of 1, 2, 5, 10, 20, 50 and 100 ng mL^−1^ for each analytes were prepared by dilutions of the mixed standard solution (20 μg mL^−1^ of each compound). Each calibration standard solution contained 10 ng mL^−1^ of ^13^C_15_-DON as internal standard, which was used for quantification of DON. Quality control (QC) samples at levels of 2, 10 and 50 ng mL^−1^ were prepared by spiking analyte-free urine with mixed standard solutions. The QC samples were included in each batch of 80 samples, and their measured values should be within ±15% of the nominal values.

### Sample preparation

Urine samples were thawed and centrifuged at 5000 × g for 15 min. Internal standard, ^13^C_15_-DON, was added to 1 mL supernatant at a final concentration of 10 ng mL^−1^, followed by a 2.5-fold dilution with phosphate buffer (75 mM, pH 6.8). A portion of 500 μL diluted sample was cleaned via Oasis® PRiME HLB μElution Plate (pre-conditioned with 200 μL methanol and 200 μL of water). After the loaded samples were slowly passed through under vacuum, the wells were washed with 200 μL of water to remove interference from urine matrix. Then the analytes were eluted twice with 100 μL each of methanol and diluted with 800 μL of water before the LC-MS/MS analysis. For the measurement of total DON and DOM-1, an enzyme digestion was added. 1 mL of the urine sample were first mixed with 1.5 mL phosphate buffer (75 mM, pH 6.8) containing 2000 Units of β-glucuronidase and incubated in a shaking water-bath at 37 °C for 18 h for digestion before the centrifugation.

### LC-MS/MS analysis

Analysis was performed on an ACQUITY UPLC™ I-Class system (Waters, MA, USA) connected to a Xevo® TQ-S tandem quadrupole mass spectrometer (Waters, MA, USA) equipped with an electrospray ionization (ESI) source. Chromatographic separation of DON biomarkers was achieved on a CORTECS™ UPLC^®^ C18 Column (2.1 × 100 mm, 1.6 μm, Waters, MA, USA). The mobile phase consisted of solvent A (water) and solvent B (methanol/acetonitrile, 80/20, v/v), running a gradient program as follow: 10% B at 0–1.5 min, 10–20% B at 1.5–1.8 min, 20% B at 1.8–7 min, 90% B at 7.1–8 min and 10% B at 8.1–9 min. The total run time was 9 min and the flow rate was set at 0.4 mL/min. The injection volume was 10 μL and the column temperature was maintained at 40 °C. MS/MS analysis in multiple reaction monitoring mode (MRM) was used to quantify DON biomarkers by reference to internal standard. Ion spray voltage was set to 3.0 kV in positive ionization mode. The MRM transitions, collision energies and cone voltages were optimized for each analyte as presented in Table 1. Other parameters were: source temperature, 150 °C; desolvation gas, nitrogen, 900 L h^−1^, 500 °C; cone gas, nitrogen, 150 L h^−1^; collision gas, argon, 0.15 mL/min.

### Method validation

Validation in terms of linearity, specificity, accuracy, precision (intra and inter-day variability) and sensitivity (LOD and LOQ) were evaluated for DON biomarkers according to the guidelines defined by the European Medicines Agency (EMEA)^29^ and US Food and Drugs Administration (FDA)^30^. The LOD (S/N = 3) and LOQ (S/N = 10) of the assay were determined using spiked urine samples at low levels. The linearity was assessed from a calibration curve on three consecutive days, using linear regression with 1/x weighting. The accuracy, expressed as the method recoveries (R_M_), as well as inter-day and intra-day precision were investigated at low (2 ng mL^−1^), medium (10 ng mL^−1^) and high (50 ng mL^−1^) spiking levels in blank urine in six replicates with internal standards correction.

### Statistical analysis

For statistical tests, undetectable DON biomarker concentration was set as half the value of their respective LOD^51,53^. The concentration data of DON biomarkers was natural log transformed for normality, prior to analyses with independent sample t-test and ANOVA to determine the differences in urinary DON levels of different subgroups (age, gender). The concentration of total DOM-1 was non-normally distributed even after logarithmically transformation. As a result, Pearson and Spearman tests were used separately to assess the correlations between total DON with free DON and total DOM-1. Statistical analysis was performed using SPSS, version 19 (SPSS, Chicago, IL, USA). A p-value <0.05 was considered as statistically significant.

### Data availability

The datasets generated during and/or analysed during the current study are available from the corresponding author on reasonable request.

## Conclusions

A high-throughput LC-MS/MS method was tested for the first time for urinary DON and DOM-1 analysis. The involvement of a 96-well μElution plate allowed the simultaneous preparation of 96 samples within 2 h, without the requirement of evaporation and reconstitution steps. The method, with significantly improved efficiency and accuracy, provides a powerful tool for large-scale population studies. With this method DON, and DOM-1 to a lesser extent, can be frequently detected in the Chinese urine samples both with and without enzyme hydrolysis. Free DOM-1 was detected at low level in a small fraction of human urine for the first time. Total DON was detected in all samples with a mean concentration at 47.6 ng mL^−1^, higher than most populations previously studied. The PDI of children and adolescents estimated based on the biomarkers levels, were higher than the adults and elders. Over 50% of the population in study exceeded the PMTDI set by JECFA, indicating a potential health risk from DON exposure.

## Electronic supplementary material

Supplementary Information
