# Supplementary material for: Risk assessment of deoxynivalenol in high-risk area of China by human biomonitoring using an improved high throughput UPLC-MS/MS method
Source: Sci Rep. 2018 Mar 1;8:3901. doi: 10.1038/s41598-018-22206-y (PMC5832810; doi:10.1038/s41598-018-22206-y)
Supplement: Supplementary file 1 — Supplementary Information [file 41598_2018_22206_MOESM1_ESM.pdf]

Supplementary Information

Risk assessment of deoxynivalenol in high-risk area of China by human biomonitoring using an improved high throughput UPLC-MS/MS method

Chunli Deng[1], Chenglong Li[1], Shuang Zhou[1], Xiaodan Wang[1], Haibin Xu[1], Dan Wang[1], Yunyun Gong[1,2]*, Michael N Routledge[3], Yunfeng Zhao[1], and Yongning Wu[1]*

[1]China National Center for Food Safety Risk Assessment, Key laboratory of Food Safety Risk Assessment, Ministry of Health, Beijing 100021, PR China

[2] School of Food Science and Nutrition, University of Leeds, UK, LS2 9JT

[3] School of Medicine, University of Leeds, UK, LS2 9JT

** Corresponding authors. E-mail: szhoupku@gmail.com (Shuang Zhou), Tel.: + 86 10 53235407, fax: + 86 10 67711813; and E-mail: y.gong@leeds.ac.uk (Yunyun Gong), Tel.: + 01 13 3431415.*

Optimization of 96-well μElution Plate

Given the complex urine matrix containing multiple endogenous water-soluble components, including mineral salts, hormones, vitamins, amino acids, urea, creatinine and other metabolites, which may cause complex interferes and column clogging, all samples were diluted 2.5-fold with phosphate buffer (75 mM, pH 6.8) prior to loading onto the μElution plate. Afterwards, to minimize the loss of DON during SPE process, only water (200 μL) was used as the washing solvent to remove salts and other water-soluble interferences. Then, elution buffers containing different amounts of methanol (20, 50, 7.5 and 100%, v/v) were tested, and only 100% methanol released all the target compounds from the sorbent. The elution volumes (50, 100, 200 and 300 μL) were also evaluated, showing that 200 μL was enough to obtain the maximum recoveries. To avoid solvent effects on chromatographic separation, the collected eluent was diluted with 800 μL of water before LC-MS/MS analysis. Following the optimized cleanup protocol, all the analytes of interest showed good recoveries ranged 88.3%~112% and acceptable matrix effects ranged 67.4%~83.2% (Table S1).

Table S1. Extraction recovery and matrix effect of each analyte.

Analyte	Extraction recovery (%)	Matrix effect (%)
DON	88.3	67.4
DOM-1	112	83.2
3-A-DON	109	82.0
15-A-DON	100	79.9

Optimization of enzymatic hydrolysis

In order to evaluate the total amount of DON and its glucuronide conjugates, an enzymatic hydrolysis using β-glucuronidase was performed. A highly contaminated urine sample (containing over 200 ng mL^{-1} of total DON) was selected from the collected samples and used for the optimization of enzyme concentration. To 1 mL of the urine sample, 1.5 mL of enzyme solution

consisting of different amounts of β-glucuronidase (100, 200, 500, 1000, 2000 and 5000 units) was added and incubated at 37 ºC for different time (8, 16 and 24 h) to check the extent of deconjugation. As shown in Figure S1, 2000 units/mL urine was sufficient for the maximum release of DON and this amount was chosen as optimal.

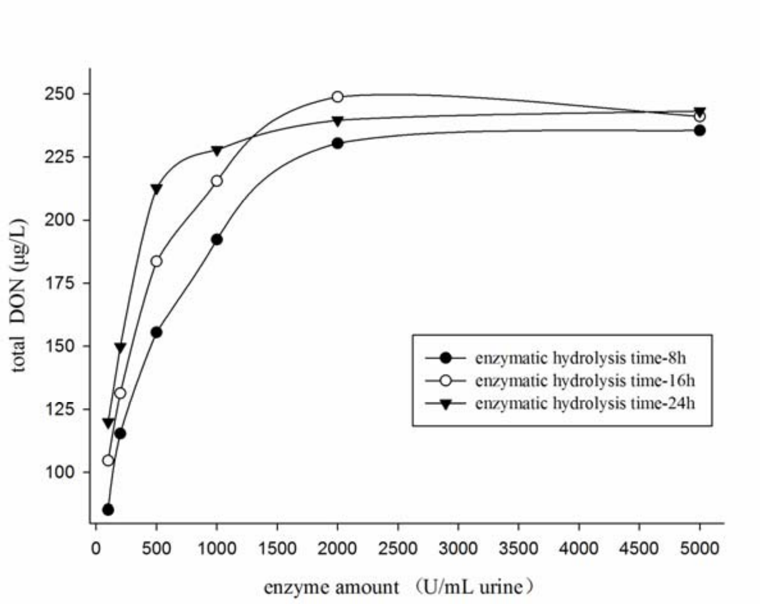

Figure S1. Effects of enzyme concentration and incubation time on the extent of deconjugation of DON-glucuronide.

Determination of 3-A-DON and 15-A-DON

Negative mode with capillary voltage of -2.8 kV was selected for 3-A-DON, 15-A-DON and ^{13}C-3-A-DON. The detailed parameters were optimized as summarized in Table S2.

Table S2. MRM transitions of DON acetylated derivatives.

Analyte	Parent ion (m/z)	Daughter ion (m/z)	Cone voltage (V)	Collision energy (eV)
3-A-DON	337.1	173.0[a]	-28	-10
		307.2	-28	-12
15-A-DON	337.1	219.1	-24	-10
		159.0[a]	-20	-14
^{13}C-3-A-DON	354.2	184.0	-30	-10
		323.2[a]	-30	-12

[a] Transition used for quantification.

Since 3-A-DON and 15-A-DON have a common precursor ion (m/z 337) and similar product

ions, chromatographic separation was necessary to avoid signal interferences and allow the identification and accurate quantification of the two compounds. To address this here, the main variables affecting chromatographic behavior were evaluated, including UPLC columns, mobile-phase components (organic modifiers, additives, etc.) and the gradient elution program. Waters CORTECS C18 UPLC column (2.1 mm×100 mm, 1.6 μm) with methanol and water as mobile phase under a gradient elution provided a complete separation of all the analytes including the position isomers 3-A-DON and 15-A-DON in 7 min for a single run.

Method validation was performed for 3-A-DON and 15-A-DON in accordance with the guidelines from EMEA and US FDA. The results in Table S3 all satisfied the acceptance criteria.

Table S3. Sensitivity, accuracy and precision for determination of 3-A-DON and 15-A-DON.

Analyte	Spiked level (ng mL^{-1})	Mean value (ng mL^{-1})	Recovery (%)	RSD_r		LOD (ng mL^{-1})	LOQ (ng mL^{-1})
				Intra-day (n=6)	Inter-day (n=18)		
3-A-DON	2	2.0	102	8.9	10.1	0.5	1
	10	10.1	101	3.0	3.6		
	50	20.0	100	1.4	2.9		
15-A-DON	2	2.1	106	8.5	14.2	1	2
	10	8.8	88	7.5	8.3		
	50	17.5	87	4.5	5.6		
